# Super-Resolution Microscopy in Studying the Structure and Function of the Cell Nucleus

**Published:** 2017

**Authors:** S. S. Ryabichko, A. N. Ibragimov, L. A. Lebedeva, E. N. Kozlov, Y. V. Shidlovskii

**Affiliations:** Institute of Gene Biology RAS, Vavilova Str. 34/5, Moscow, 119334, Russia; I.M. Sechenov First Moscow State Medical University, Trubetskaya Str. 8, bldg. 2, Moscow, 119048 , Russia

**Keywords:** histone, DNA, super-resolution microscopy, chromatin, chromosome, cell nucleus

## Abstract

In recent decades, novel microscopic methods commonly referred to as super- resolution microscopy have been developed. These methods enable the visualization of a cell with a resolution of up to 10 nm. The application of these methods is of great interest in studying the structure and function of the cell nucleus. The review describes the main achievements in this field.

## INTRODUCTION

The cell nucleus performs the cardinal function of storing and processing hereditary information. For a long time, progress in nuclear structure and function research has been hampered by the lack of adequate techniques to explore intranuclear structures. Today, the most popular method used for studying the nuclear structure is scanning confocal microscopy, which has a resolution limit of about 200 and 500 nm (lateral and axial resolution, respectively). Researchers also use other microscopic techniques that enable them to explore nuclear processes at the molecular level [1]. However, studying the nucleus in a range of 20–200 nm remains technically arduous. Only the development of 3C methods and introduction of the Hi-C method improved understanding of the principles of chromatin organization at this level [2, 3]. In recent decades, another approach, known as super-resolution microscopy (SRM), has been actively advancing and has enabled researchers to achieve a breakthrough in the exploration of the structure and function of the cell nucleus. In SRM, the issue of the diffraction limit is overcome by the use of various technologies, including both technical solutions in the microscope design and computational methods for image reconstruction [4].

## THE PRINCIPLES AND CAPABILITIES OF SRM

The most significant advances in super-resolution imaging were achieved using far-field microscopy [5]. 4Pi microscopy uses two opposing objective lenses focused to the same point, which improves axial resolution to 100 nm. In other SRM methods, the diffraction limit is overcome in two ways: The first one is spatial and/or temporal modulation of the transition between two molecular states of a fluorophore, and the second one is a narrowing of the point spread function of an ensemble image of many fluorophores located near each other. The main methods of the first group include stimulated emission depletion (STED), ground state depletion (GSD), structured illumination microscopy (SIM), and some of their combinations with I5M microscopy (4Pi variant) [6, 7]. The main methods of the second group are photoactivated localization microscopy (PALM), fluorescence photoactivation localization microscopy (FPALM), stochastic optical reconstruction microscopy (STORM), and binding-activated localization microscopy (BALM) [7, 8].

The SIM technique uses specifically structured illumination of the object, which doubles the resolution on each axis and provides 100 nm for lateral resolution and 300 nm for axial resolution. In 3D, the resolution increases eightfold. Frequent use of this microscopy is related to the possibility of preparing samples in a standard way and to apply standard fluorophores. In STED, the sample is simultaneously illuminated by two lasers at different wavelengths, exciting and depleting; this combination of lasers excites fluorescence in a narrow region of a focal spot 50–80 nm in size. This method achieves a resolution of 20 nm in the focal plane and 45 nm in all three directions.

PALM, FPALM, BALM, and STORM belong to single-molecule localization microscopy (SMLM). The sample is excited multiple times with weak laser pulses. Each pulse activates and precisely localizes (based on the point spread function) a small fraction of fluorophores. The image resolution in these methods depends on the ratio of the photon number from an individual fluorophore to the total fluorescence background and, in theory, can reach 1 nm [9].

More detailed information on the principles of these methods, as well as their advantages and disadvantages, can be found in recent reviews [10, 11]. One of the latest SRM techniques is W-4PiSMSN that enables visualization of the whole cell with a resolution of 10– 20 nm, with the cell thickness attaining 10 μm [12].

Global nuclear structure is usually visualized via the labeling of histones with fluorescent proteins [13] or binding of fluorescent labels to DNA and RNA [14-16]. Histones can be fused to fluorescent proteins and, also, chemically modified [17, 18]. In SRM experiments, DNA can be labeled with classical dyes, such as Hoechst and DAPI [19]; also, more photostable and photoswitchable fluorophores have been developed [15, 20, 21]. SRM is compatible with click chemistry, which enables the introduction of fluorescent labels into DNA [22, 23]. A technique based on intrinsic nucleotide fluorescence was proposed for the visualization of DNA in [24].

In SRM, specific DNA sequences can be detected using classical fluorescence *in situ *hybridization (FISH) that preserves, in general, the fine nuclear structure [25]. The DNA-PAINT method has been proposed. It enables multicolor imaging with a resolution of more than 10 nm [26]. What’s more, methods of multiplexed FISH with probe exchange have also been put forth [27, 28]. Specific DNA sequences can also be localized with programmable gRNA/dCas9 complexes [29].

In SRM-based DNA visualization, the resolution usually hovers around 25 nm, which corresponds to about 70 bp linear double-stranded DNA [30, 31]. A resolution of 70 nm has been achieved in living cells [32]. At present, a resolution of 5 nm in densely packed samples is achievable using oligonucleotide hybridization [33]. Therefore, SRM has opened perspectives for high-resolution optical genome mapping [34]. For example, DNA repeats in the Yq12 heterochromatin region of the human genome have been studied [35]. The number of trinucleotide repeats in the 5’-untranslated region of the *FMR1 *gene has been counted [36]. Single-molecule microscopy combined with Oligopaint probes can help researchers detect single-nucleotide polymorphisms and, thus, distinguish between homologous chromosomes [37]. A complementary method of molecular beacons has been proposed, which enabled the visualization of a 2.5 kb unique DNA sequence [38]. SRM allows one to very accurately measure the volume occupied by individual gene loci in the nuclear space, evaluate their level of compactification [39], and detect the exact spatial relationships between individual loci [40, 41]. Also, epigenetic markers can be very accurately localized in the nucleus [42, 43].

A protein that ends up being detected in the nucleus by SRM is usually synthesized in the cell in fusion form with another fluorescent protein. It is important to note that SRM allows one not only to localize a fluorophore, but also to determine its amount at the localization site with an accuracy of up to one molecule [44-46]. There are methods that allow for simultaneous detection of several proteins (currently, up to nine) in a single sample [47, 48].

SRM has been used in many studies. Below are the main results obtained using the method when exploring the structure and functions of the cell nucleus.

## GENERAL ARCHITECTURE OF THE CELL NUCLEUS AND CHROMATIN PACKAGING

The general nuclear architecture has been studied with high resolution in different cell types [49]. For example, there are reports on the reorganization of the cell nucleus structure during myelopoiesis in humans [50], neurogenesis in mice [51], in cells of early embryos and cloned bovine cells [52, 53], and in the cell cycle of yeast [54]. Such studies are often descriptive, because very little is known about the mechanisms of fine nuclear structure formation.

In one of the early studies, the fine organization of the mammalian cell nucleus was explored using 3D-SIM [55]. In the nucleoplasm, channels and lacunas (interchromatin compartment) starting at the nuclear pore and expanding throughout the nuclear space were revealed. Within each chromosome territory, internal regions of higher order chromatin domains are separated from interchromatin by a 100- to 200-nm-thick layer of decondensed transcriptionally active chromatin (peripheral chromatin). The latter is enriched in markers of active transcription and replication, while splicing markers are predominantly localized in the interchromatin compartment. Clusters of RNA polymerase II were revealed, with a significant fraction of transcription being observed outside the clusters. The SRM and 3C data enabled researchers to generate a nuclear organization model where active chromatin forms clusters and aligns at the boundaries of the network of the intracellular channels pervading most of inactive chromatin [56]. The localization of transcription regulation sites at the periphery of chromatin domains was confirmed by FISH [57].

Exploration of the nuclear periphery in human cells using SIM demonstrated that the number of heterochromatin markers is generally reduced near nuclear pores, but both euchromatin and heterochromatin may form contacts with the pore. Chromatin-modifying enzymes are associated with nuclear pores, which may be indicative of the pore’s role in the organization of the nuclear chromatin structure [58].

One of the first studies that used labeled histones demonstrated that the total histone density is different depending on the type of human cells. Global fluctuations in the nuclear histone density on a scale of 1–2 μm were detected using dSTORM [59]. The total histone H2B density in the nuclei of a human U2OS cell line measured by PALM was used to determine the parameters of a nuclear chromatin distribution model and, also, to confirm the fractal globule model [60]. A study of the histone H2B dynamics in HeLa cells using dSTORM showed that histones form clusters that are separated by a distance of about 100 nm and move at a rate of 3 nm/s in the interphase nucleus [18]. In human cells, histone clusters with a mean diameter of 160 nm have been observed using PALM. The formation of clusters was shown to depend on cohesin and internucleosomal interactions, but not on transcription. These domains are present both in mitotic chromosomes and in the interphase nucleus. Perhaps, they are the building blocks of chromosomes [61].

Using PALM, filaments with a diameter of 70 nm were shown to be present in metaphase chromatin in Drosophila [62]. The use of STED microscopy to study the chromatin structure in cardiomyocytes revealed 40–70 nm chromatin domains [63]. In mouse and human cells, the nucleosome distribution in DNA has been analyzed in detail using STORM [64]. Nucleosomes were shown to form heterogeneous, different-size groups (clutches) in the nucleus. The mean number of nucleosomes in a group and their density depend on the cell type: in pluripotent cells, these groups are less dense and contain less nucleosomes. RNA polymerase II is predominantly associated with the smallest clutches, while histone H1 and heterochromatin are rich in the largest clutches.

A large-scale study of DNA packing in various chromatin types in Drosophila cells was performed using 3D-STORM [65]. Transcriptionally active, inactive, and Polycomb domains were studied. The former were the least compact, while the last were the densest. The packing degree of these domains varied depending on the domain size: the larger the active domain size, the less dense the domain, whereas the larger the Polycomb domain size, the higher its density. Polycomb domains, unlike the other two types of domains, were demonstrated to be characterized by a high degree of DNA intermixing within the domain and almost complete absence of intermixing with neighboring domains of a different type. Models of chromatin organization domains of different types have been proposed.

Thus, intranuclear chromatin domains differing in size have been described to date. Further research is required to clarify the correlation between intranuclear domains themselves and with the topologically associated domains detected by Hi-C analysis.

## HETEROCHROMATIN

The structural details of satellite DNA heterochromatin in aging human cells have been studied using STED microscopy [66]. In both aging and dividing cells, satellite DNA is packed into a set of compact globules separated by linkers. But during aging, the distance between the globules increases. Light-sheet Bayesian super-resolution microscopy (LSBM) helped reveal that the major heterochromatin protein HP1 forms a network in human nuclei [67].

A SIM study demonstrated that, in the interphase, transcriptionally inactive chromatin in yeast cells is less compact than euchromatin. At the same time, highly condensed bodies spanning 50 kb in sequence and flanking inactive telomeric regions were found. The formation of these bodies was independent of the HP1 protein, but it depended on methylation of the H3K36 residue [68].

3D-STORM combined with Oligopaint probes was used to visualize chromatin associated with the Polycomb factor in embryonic stem cells and mouse neuronal progenitor cells [69]. The formation of compact regions in the *Hox *gene locus was established; in this case, removal of the Phc1 protein led to chromatin decompaction. These regions consist of small, discrete domains containing 20–140 kb DNA, which differ from topologically associated domains (TADs). Another study revealed several hundred Polycomb clusters in the nuclei of Drosophila cells; the clusters were different from previously studied Polycomb bodies. The number of clusters was dependent on the integrity of the polymerizable SAM-motif of the Ph protein and its content in the cell. It is probable that these clusters form a network of long-range interactions throughout the genome, thereby maintaining the global nuclear architecture [70].

The structure of Barr bodies in mice cells was determined using 3D-SIM [71]. Despite a significant decrease in size, an inactivated X chromosome retained the general structure characteristic of normal chromosome territory. But in this case, the volume of the internal channels was significantly smaller. The same technique was used to determine the localization of the RNA-binding proteins Rbm15, Spen, and Wtap in Xist RNA [72]. A STORM analysis of an inactivated X chromosome in mouse fibroblasts revealed that only 50–100 Xist molecules and about 50 clusters of the PRC2 complex are present on the X chromosome [73].

## STRUCTURE OF CONDENSED CHROMOSOMES

Chromatin is located not only along a chromosome (bands on polytene chromosomes), but also radially. A peripheral localization of actively transcribed loci, beginning with the prophase, on the mitotic X chromosome of Drosophila males was demonstrated using SIM. In general, in a condensed mitotic chromosome, silent regions are localized closer to the chromosome’s axis, while active regions are closer to the surface [74]. 3D-SIM was used to study the differential accessibility of the different loci of a metaphase chromosome in human lymphoblasts [75].

Similarly, SMLM-based visualization of mouse chromosomes at the pachytene stage allowed researcher to identify three chromatin types [76]. The first one is located radially (it carries the active transcription marker H3K4me3) and forms loop-like structures. The second is located along the chromosome axis and carries the H3K27me3 marker. Finally, there is centromeric chromatin carrying the H3K9me3 marker.

3D-SIM was used to investigate the involvement of various condensin forms in chromosome scaffold formation in chicken cells. In condensin I subunit knockout cells, mitotic chromosomes had become shorter and wider and had a diffuse scaffold; in condensin II subunit knockout cells, the scaffold was more defined and the chromosomes were more stretched and lacked axial rigidity [77]. The spatial organization of a meiotic chromosome was studied using various SRM methods [78]. In yeast cells, 3D-SIM demonstrated the formation of a chromosome axis from meiotic cohesin, as well as impairment in the chromosome structure in the absence of cohesin [79, 80].

The so-called T-loops were visualized on mouse telomeres using STORM. The investigation of the loop structure in the setting of various mutations showed the importance of the TRF2 factor in the formation of this structure [81].

Single-molecule microscopy was used to study centromere organization in chicken cells: centromeric chromatin in the chicken was represented by layers with alternating domains enriched in CENP-A or H3 histones. During mitosis, the CENP-C-dependent mechanism links CENP-A-blocks [82]. In yeast cells, PALM was used to count CENP-A molecules in the centromere; the histone was shown to have accumulated at the centromere in the G2 phase in yeast, unlike metazoan cells [83].

As was shown with SIM in the cells of various plant species, histone CENH_3_ and modified histone H2AThr120P are incorporated into various nucleosomes that form different domains [84]. In barley, two centromeric histone variants – alpha- and beta- CENH_3_ – are incorporated into various domains of centromeric chromatin in the interphase, with the incorporation pattern being tissue-specific [85].

## TRANSCRIPTION

SRM was used to visualize the processes occurring during the activation of gene transcription. STORM imaging revealed that, upon activation of the mammalian *Hoxd *gene, its locus becomes de-compacted and acquires an elongated configuration [86]. In mouse cells, the beta-globin locus was visualized using 3D-SIM: the inactive locus had several different conformations; during the differentiation of the cells, the locus decreased in size and its structure became more arranged [87]. Local de-condensation of chromatin in active transcription loci was visualized using BALM in chromatin spreads from HeLa cells. Stimulation of the cells led to the appearance of open chromatin regions of about 388 nm in length and about 60 nm in width enriched in the active form of RNA polymerase II [88]. Using the same technique, long-range contacts forming in the genome of an individual cell with the participation of the transcription factors YAP, SRF, and NF-kappaB [89] were visualized.

Single-molecule microscopy, combined with light sheet microscopy, enabled us to perform a quantitative analysis of transcription factories in mammalian cells [90]. More than 70% of the transcription sites were found to contain only one RNA polymerase II molecule, which contradicts the existing models. The dynamics of transcription factories has been studied [31, 91]: these factories are not static structures and can form in the nucleus upon stimulation of the cell. Formation of factories is not blocked by the inhibitors of RNA polymerase II elongation; i.e. the factories form at the transcription initiation stage.

The concept of transcription on an immobilized transcription factory was confirmed using SRM. Induction of human cells with a cytokine leads to closer approximation of two genes in the nucleus, which are remotely located in the genome, with their transcripts also being located near each other [92]. In the same model, the process of induced transcription on a 221-kb-long gene template was visualized [93]. The transcription factory initially associates with the gene promoter, and then the DNA template is pulled through this transcription factory. In this case, the promoter remains near RNA polymerase for a while, but then it can lose contact, and re-initiation can occur in another factory.

Visualization of transcription on lampbrush chromosomes using dSTORM enabled the imaging of newly synthesized RNA packaging. The splicing and tight packing of mRNA result in the fact that the thickness of a transcribed chromatin loop remains almost unchanged along the active gene [94].

In plants, a study of the distribution of different RNA polymerases II using SIM and PALM revealed networks of these molecules in euchromatin, with different forms constituting different networks [95, 96]. An increased number of polymerase molecules in the nucleus of polyploid cells was found, which was in general proportional to the total number of genes. Probably, plants have another form of transcription organization, different from that in mammalian transcription factories.

The localization of transcription factors in the nucleus has been primarily studied using SMLM. The binding sites of the Sox2 transcription factor in mice are found to form clusters in the nucleus, which plays an important role in the regulation of the transcription of its target genes [97]. Similarly, the human STAT1 factor forms clusters, with their size and number increasing significantly during transition from the G1 to the G2 phase of the cell cycle, as well as upon stimulation of the cell with cytokines [98]. The transcription factor FoxP3 in T cells forms two types of complexes with other factors: the activation complex located closer to the nucleus center and the repression complex located at the nuclear periphery [99]. SRM was used to study the distribution of histone H2A and the chromatin remodeling factor Snf2H subunit [100], as well as the euchromatin protein MAD2L2 [101].

4Pi-microscopy was used to study the internal structure of PML bodies in human cells. The transcription factors Sp100 and PML form a 50- to 100-nm-thick shell of bodies, which is permeable to proteins. The inner region of the bodies contains polymeric chains of the SUMO protein, which serves to concentrate SUMO-binding factors in the bodies [102].

Exploration of the nucleolus in human cells using SMLM showed that, like the nucleus, it has a very heterogeneous structure: the nucleolus contains regions with high and low concentrations of newly synthesized RNAs [103].

## REPLICATION

The structure of replication factories in human cells was studied using STEM microscopy with labeled PCNA and RPA proteins [104]. The diameter of the replication factories was found to be below 160 nm, on average. In the early S phase, up to 1,400 factories with two or three replication forks each were detected. The factory size in mouse cells, estimated using SIM and SMI, was 125 nm [105]. In mammalian cells, about 5,000 replication factories, with each of them being a separate replication site, were detected using SIM in the S phase [106].

A study of replication in yeast cells using SIM revealed that clustering of replicons in single replication factories was a stochastic process and varied greatly from cell to cell, but once associated in one factory, replicons remained stably linked [107]. As demonstrated using SIM, replication of peripheral heterochromatin in mammalian cells, which was tightly associated with the lamina, occurred without disassembly of the latter [108].

SRM helped to reveal a new function for the replication factor Cdt1 in the formation of an extended conformation of the kinetochore complex Ndc80 and its stable association with microtubules in mitotic human cells [109]. Data on the location of kinetochore components of a Drosophila chromosome and on the role of the Spc105 protein in the assembly of this structure were obtained [110]. Also, the kinetochore structure in yeast [111] and human [112] cells was studied. Visualization of mitosis enabled us to track the switching dynamics of the directional movement of sister kinetochores in the metaphase [113].

Coordination of replication and transcription was studied in mammalian cells [114]. In nucleoli where the rRNA genes are actively transcribed, there is a strong negative correlation between these processes. At the same time, this correlation is absent in the nucleoplasm.

## REPAIR AND RECOMBINATION

The repair process was also studied using SRM [115]. The localization pattern of histone gamma-H2AX (a double-strand DNA break marker) and the dynamics of its localization sites have been explored in a number of studies using different methods [116-118]. Also, the mutual arrangement of gamma-H2AX and the Ku repair complex was studied, and the number of molecules in this complex at the repair site was counted [119]. dSTORM was used to elucidate the molecular basis of nonhomologous end joining in human cells: it was found that DNA ends first interact with each other through protein filaments, then the two ends position themselves relative to one another, and ligation occurs [120].

As shown using 3D-SIM in HeLa cells, the pattern of the proteins BRCA1 and 53BP1 inside repair foci was mutually exclusive. Probably, their mutual arrangement determines the choice of a repair pathway [121]. STED-microscopy was used to visualize the repair factors gamma-H2AX, 53BP1, and Rad51 in HeLa cells after exposure to ionizing radiation. The first two proteins form regions whose size depends on the radiation energy (540 nm for strong and 412 nm for weaker radiation); furthermore, these regions display an internal structure and a negative correlation in the distribution of the two proteins. Rad51 forms regions that lack an internal structure, and their size (135 nm) is independent of the radiation energy [122]. As revealed by dSTORM in human cells, the partners BRCA2 and Rad51 are localized in different sites of the repaired DNA, which indicates different dynamics of their interaction with DNA [123]. As shown using STED and 3D-SIM in human cells, the gamma-H2AX break marker is distributed over nearby chromatin loops, and this process is controlled by the CTCF protein. Therefore, the repair focus is a close group of nano-foci, with each of them being a chromatin loop [124].

The synaptonemal complex formed between homologous chromosomes during meiosis was visualized and studied using various SRM methods in barley and wheat [125, 126], mouse [127], *Caenorhabditis elegans* [128], and yeast [129] cells.

## NUCLEAR MEMBRANE

The nuclear membrane contains a variety of transmembrane proteins. For their localization, SRM methods with an axial resolution of more than 10 nm have been developed [130, 131].

The structure of the most important nuclear periphery component, the nuclear pore, has been studied with high accuracy using various SRM methods. In different species, the pore size [132] and the position of its main subunits [133, 134] have been determined, and the pore assembly process has been visualized [135]. Accumulation of several thousand images of individual nuclear pores has enabled a structural analysis of the pore with an accuracy of more than 1 nm [136]. The localization and distribution of individual nuclear pore subunits over the membrane have been determined [137-141]. Using SRM, the contacts between nucleoporins and transport receptors have been studied [142], and contact between the nuclear pore and the active locus has been demonstrated [143].

SPEED microscopy with a spatial resolution of 8 nm and a temporal resolution of 2 ms was used to trace transport through the nuclear pore in human cells [144]. Only 36% of mRNA molecules entering the pore were shown to be successfully exported to the cytoplasm, with a transport time of about 12 ms. The kinetics of mRNA transport through nuclear pores in mouse cells was also studied with a spatial resolution of 26 nm and a temporal resolution of 20 ms [145]. Export was shown to be a three-stage process, including docking to the pore (80 ms), transport (5–20 ms), and release (80 ms) of the transcript.

The investigation of the nuclear periphery demonstrated that there are invaginations in the lamina, which had been indistinguishable using confocal microscopy [146]. Invaginations have been described in the interphase nucleus in different cell types, but their functions remain unclear. They may be involved in mRNA transport [147].

SIM microscopy has enabled an investigation of the relationship between the nuclear lamina and the actin cytoskeleton in human cells. Mechanical pressure of the actin filament has been found to cause the formation of invaginations in the lamina and the emergence of condensed chromatin domains [148].

## CONCLUSION

The topicality of light microscopy for the investigation of intranuclear processes has always been high. An intensively developing group of SRM methods opens new perspectives in this field [149]. Theoretically, SRM may achieve a resolution of up to 1 nm: therefore, it is a unique tool to be used to study processes on a scale ranging from an individual molecule to a whole cell. Apart from helping solve basic problems, SRM is applied in the study of processes that occur in cell nuclei in various pathologies. SRM has been used to study Hutchinson-Gilford progeria [150], Alzheimer’s disease [151], hypoxia and fasting in cardiomyocytes, oncogenesis [21, 152], and viral infections [150, 151]. SRM-based techniques have been developed for the diagnosis of diseases, in particular cancers [153].

The data presented in this review demonstrate that SRM has significantly expanded our knowledge about how the nucleus functions at various levels of its organization. At the same time, it is obvious how little the potential of this powerful method has been used to date. Undoubtedly, the combination of SRM with other modern methods is poised to become the basis for new discoveries in the biology of the cell nucleus in the near future.

## Abbreviations

SRM: super-resolution microscopy
BALM: binding-activated localization microscopy
FISH: fluorescence in situ hybridization
PALM: photoactivated localization microscopy
SIM: structured illumination microscopy
SMLM: single-molecule localization microscopy
STED: stimulated emission depletion
STORM: stochastic optical reconstruction microscopy
